# Beliefs and social behavior in a multi-period ultimatum game

**DOI:** 10.3389/fnbeh.2015.00029

**Published:** 2015-02-13

**Authors:** Ofer H. Azar, Yaron Lahav, Alisa Voslinsky

**Affiliations:** Department of Business Administration, Ben-Gurion University of the NegevBeer Sheva, Israel

**Keywords:** ultimatum game, social preferences, beliefs, experimental economics, behavioral economics

## Abstract

We conduct a multi-period ultimatum game in which we elicit players' beliefs. Responders do not predict accurately the amount that will be offered to them, and do not get better in their predictions over time. At the individual level we see some effect of the mistake in expectations in the previous period on the responder's expectation about the offer in the current period, but this effect is relatively small. The proposers' beliefs about the minimum amount that responders will accept is significantly higher than the minimum amount responders believe will be accepted by other responders. The proposer's belief about the minimal acceptable offer does not change following a rejection. Nevertheless, the proposer's offer in the next period does increase following a rejection. The probability of rejection increases when the responder has higher expectations about the amount that will be offered to him or higher beliefs about the minimal amount that other responders will accept.

## Introduction

In experiments on social preferences involving real monetary outcomes, the results frequently reveal that people are motivated not only by their own outcomes but also by those of others, perceptions of social norms, and beliefs. In interdependent situations such as simple bargaining environments people are even willing to reduce their payoff in order to punish those who mistreat them. Insights about the motivations and characteristics of non-selfish behavior obtained from experiments may help to understand a variety of economic behaviors. Notably, subjects' desire to reduce differences between theirs and others' payoffs affects their decisions. They also want to increase or decrease others' payoffs depending on how fairly those others are behaving. Furthermore, people's perceptions regarding themselves and their beliefs about others might affect their social behavior.

In the ultimatum game (Güth et al., 1982), a first player (proposer) offers a split of the bargaining pie to a second player (responder), who can either accept the offered split or reject it. A rejection results in a payoff of zero to both players. Therefore the subgame perfect Nash equilibrium (SPNE) involves a responder who accepts any positive offer and consequently the proposer will offer the minimal positive amount^1^. However, actual play in experiments is inconsistent with this prediction: responders who feel they have been treated unfairly (receiving low offers) often harm the proposer's payoff even at a cost to themselves by rejecting positive offers^2^. Moreover, proposers usually anticipate this and therefore offer much above the minimal positive amount. The responders' rejection of low offers suggests that social motivations such as fairness are important. It is harder to know whether the proposers' behavior of offering significant amounts is only strategic (to avoid rejection) or also reflects social considerations. Comparison of ultimatum games to other games such as the dictator game, in which the first player determines a division of the pie and the second player cannot reject it, suggests that both strategic and social motivations affect the proposer's behavior in the ultimatum game^3^. Brañas-Garza et al. (2014), for example, use the combination of behavior in the dictator game and the ultimatum game to classify subjects into two different sub-populations, of prosocial punishers and spiteful (antisocial) punishers.

Previous findings in ultimatum games show that when an unequal share of roughly a quarter of the pie is proposed, the responders' sense of fairness is often violated, and consequently, approximately one half of the offers are rejected. As Güth et al. (1982) show, relative to the theoretical predictions, proposers offer far too much and responders reject far too frequently. However, there is some variance in the rejection portion based on multiple moderating variables such as gender, repetition, players' level of anonymity, etc. (Camerer, 2003). Several studies on ultimatum games with high stakes find that while responders may reject less often when the stakes are high, they still reject some positive offers (Hoffman et al., 1996a; Slonim and Roth, 1998; Cameron, 1999).

Güth (1988) and Straub and Murnighan (1995) suggest that players want to be treated fairly. Responders expect to receive equal offers, and therefore reject small offers in order to punish proposers who behave unfairly. Proposers are also influenced by notions of fairness, and consequently make equal offers. In contrast, Weg and Zwick (1994) argue that proposers realize the risk of rejection of small offers, and therefore maximize their expected profits by offering approximately half of the pie. Other studies show that behavior can be affected by the context and the beliefs of the players. For example, when the assignment of players to become the proposers is made because they obtained a higher score on a knowledge test, they offer less fair offers (Hoffman et al., 1994). Responders who are unaware of the size of the divided amount accept smaller amounts, and proposers who know about the unawareness of the responders offer less (Pillutla and Murnighan, 1995; Straub and Murnighan, 1995; Croson, 1996; Kagel et al., 1996). Other experiments show that when proposers feel more anonymous, they propose less (Hoffman et al., 1996b; Novak et al., 2000). Finally, if proposers know that their decision can be observed by others, they become more generous (Bolton and Zwick, 1995; Straub and Murnighan, 1995).

Belief elicitation is the task of capturing individuals' thoughts about future outcomes. Understanding individuals' beliefs may help to predict their behavior and decisions. Haruvy et al. (2007) elicit traders' predictions of future price trajectories in repeated experimental asset markets and show that traders' expectations are influenced by previous prices. Isen et al. (2010) present the possibility of predicting behavior in economic games by asking the players about their beliefs and intentions. They ask players directly about their sense of fairness, defined as what they think they ought to do. Gächter and Renner (2010) elicit beliefs in public goods experiments and show that when beliefs are elicited, they affect decisions. Armantier and Treich (2013) combine theory and experimental evidence to address the issue of proper scoring rules used to incentivize belief elicitation. Their results show complex distortions of reported beliefs, questioning the ability of proper scoring rules to identify truthful beliefs. Interestingly, Haruvy et al. (2007) report no effect of belief elicitation on outcomes.

Behavior is often affected by beliefs about the opponents' actions, and can be a response to intentions and not only to outcomes. Rabin (1993), for example, assumes that when choosing a strategy, each player's subjective expected utility depends not only on their strategy, but also on higher order beliefs (their beliefs about other players' strategy choice, and their beliefs about the beliefs of others about their own strategy). Falk and Fischbacher (2006) present, in light of a wide range of experimental games, a formal theory of reciprocity, where people evaluate the kindness of an action not only by its consequences but also by its underlying intention. Loewenstein (2000) argues that visceral factors (such as emotions, feelings and drive states) have important—though often underappreciated—consequences for beliefs and behavior. Charness and Rabin (2002) design a range of simple experimental games, showing that subjects behave according to their beliefs. Falk et al. (2008) provide experimental evidence for the behavioral relevance of fairness intentions.

Offerman et al. (1996) present an experimental analysis of contributions and measure of individuals' expectations about the behavior of others in their group. Dufwenberg and Gneezy (2000) measure beliefs experimentally and find positive correlation between a given sum of money and the giver's expectations about the responder's expectations concerning this sum. Brañas-Garza and Espinosa (2011) find that subjects do not predict end-game effects in a linear public good game, and there is very little updating of beliefs. Danz et al. (2012) study beliefs and actions in a repeated normal-form game and find consistency between beliefs and actions. Ziegelmeyer et al. (2010) find that participants' elicited beliefs are consistent with their own behavior. Interestingly, Costa-Gomes and Weizsacker (2008) find in one-shot games that subjects act inconsistently with their stated beliefs in almost half of the games.

Beliefs are also elicited in dictator games. Rigdon and Levine (2011) elicit beliefs in a modified dictator game to better understand gender differences in decisions. They show that the behavioral differences between males and females is a result of differences in beliefs, rather than in the cost of giving. Iriberri and Rey-Biel (2013) elicited deciders' beliefs about the preferences of other deciders, finding correlations between subjects' beliefs and their social type. Brañas-Garza and Rodriguez-Lara (2014) also elicit participants' beliefs of their peers. They find that while dictators believe they are more generous to their recipients than other dictators, recipients believe that their dictators are less generous than others. While it is interesting to see how beliefs are formed in the dictator game, it cannot provide insights on the connection between experience, beliefs and behavior. A dictator may have beliefs about her actions, but there is no feedback to learn from, because there is no behavioral reply on the side of the recipient.

We use the ultimatum game, where the proposer may experience a rejection to her offer, and where responders may feel disappointed. This type of feedback may affect both belief formation and decisions in the future. The ultimatum game can therefore enrich our understanding of social preferences. Camerer (2003) describes the ultimatum game as a “… crisp way to measure social preferences rather than a deep test of strategic thinking.” Responders may find an offer either insulting or fair. Proposers, on the other hand, may find rejections unfair or understandable.

The importance of the ultimatum game as a tool for studying social behavior and the significance of beliefs in affecting behavior lead to the question what may we learn by eliciting beliefs in the ultimatum game. In this article we study the formation of beliefs and their connection with social behavior in the ultimatum game. We explore whether actions taken by subjects are sometimes inconsistent with their stated beliefs. We also study whether beliefs of players in one role are consistent with beliefs and actions of players in another role. Finally, we also test how past experience affects the formation of current beliefs and how they, in turn, affect future decisions. Because the experiment involves five periods, we can also analyze how beliefs and actions develop over time.

## Experimental design and research hypotheses

Seven experimental sessions were conducted in the computer lab of the Guilford Glazer Faculty of Business & Management at Ben-Gurion University of the Negev, with a total of 68 participants (29 female and 39 male)^4^. Each session was formed with an even number of participants (between 8 and 12). All participants were undergraduate students recruited by posting announcements on an electronic university-wide message board, in a variety of faculties and disciplines. The computers were programmed using the z-tree software (Fischbacher, 2007) and no individual participated in more than one session.

The sessions were structured as follows: subjects were randomly assigned either the role of a proposer or the role of a responder. Then, subjects read the instructions, followed by one trial period and a short multiple-choice questions quiz to make sure they understood the instructions. Then, each subject played an ultimatum game five times (referred to as “periods”), and remained in all periods in the same role. In each period, one proposer was randomly matched with one responder, to avoid learning of a specific opponent's behavior or reputation building. The participants could not know who their opponents were^5^.

The proposers were first asked in each period to make an offer to responders on how to divide between the two of them a pie of 50 Shekels (about 14 USD). After making the offer, each proposer submitted her belief regarding the minimum amount the responder will be willing to accept. Responders were asked at the beginning of each period to submit their beliefs regarding the amount they expect to receive from the proposer, and the minimal amount they think that other responders will be willing to accept. Proposers did not know that responders' beliefs are elicited and vice versa. Later they were presented with the offer made by the proposer and were asked to decide whether to accept or reject it. This experimental design allowed us to explore various issues related to behavior, beliefs, and how these develop with experience.

In each period, responders observed the amounts offered by their proposers, and proposers could see if their offer was rejected or accepted. At the end of each period, they could observe their payoff. No additional feedback was given to the participants. At the end of the session, the computer randomly chose one period and the results from this period were used for compensation. This type of payoff eliminates wealth effects, which are not being studied in this paper. We did not pay the participants for their beliefs. While understanding the importance of such element, we could not implement payment incentives to most of the beliefs we elicit. Paying for belief elicitation should be based on a scoring rule, which scores the assessor (in our case, participants) based on how close they were to some actual outcome^6^. Two of the three types of elicited beliefs were not based on such cases. Proposers' beliefs about the minimal amount that responders will accept cannot be compared to a real outcome, because we do not know this minimal amount, but rather only whether a specific offer was accepted or rejected. The same issue applies to responders' beliefs about the minimal amount that other responders will accept. Regarding responders' beliefs about the amount they expect proposers to give, any payment may increase responders' tendency to reject offers that are lower than expected, because responders may consider payment from belief elicitation as some sort of compensation^7^.

Let us turn to explain our main hypotheses. While much of the literature finds actions to be consistent with beliefs, some examples for inconsistency exist (e.g., Costa-Gomes and Weizsacker, 2008). We hypothesize that proposers can easily realize that it is not beneficial to offer amounts that they believe will be rejected, and consequently that proposers will offer at least the minimal amount that they believe is required for acceptance of their offer:

*H1: Proposers will not offer to responders amounts below the minimum they think that responders will accept*.

Subjects in the experiment were assigned randomly to the roles of proposers and responders. It therefore seems natural to expect that responders will be able to imagine themselves in the role of proposers, and consequently to make correct predictions about the offers that proposers will make. However, it is also possible that responders will manage their expectations strategically, and in particular they may be pessimistic and expect a relatively small offer, in order to experience later a positive surprising gift when the actual offer is received (see Khalmetski et al., 2013).

*H2: Responders' expectations about the amounts that will be offered to them will be on average either equal to or lower than actual offers*.

Proposers are asked to evaluate the minimal offer that will be accepted, and similarly responders are asked about the minimal offer that other responders will accept. Similar to the previous hypothesis, because proposers and responders come from the same subject pool and role assignment is random, it seems natural to expect that their beliefs will be similar. Brañas-Garza and Rodriguez-Lara (2014), for example, find no differences in expectations between dictators and recipients in dictator games, suggesting that the role in the experiment might not affect the formation of beliefs. To test this, we hypothesize as follows:

*H3: Proposers' beliefs about the minimal acceptable offer will be on average equal to responders' beliefs about the minimal acceptable offer of other responders*.

Proposers whose offers are rejected may infer that they underestimated the minimal acceptable amount and therefore have to raise their offers so that they get accepted^8^. This leads to the next double hypothesis:

*H4a: After a rejected offer, the proposer's belief about the minimal acceptable offer will increase*.

*H4b: After a rejected offer, the proposer's offer will increase*.

The ultimatum game is not something people face much in the real world and therefore the responders' expectations about the offers they will receive are likely to be updated during the experiment according to the offers received (despite changing the opponent in each period). This leads to our next hypothesis:

*H5: The change between periods in the responder's expectation about the offer is an increasing function of the difference in the previous period between the actual offer and the responder's expectation*.

Rejection of any positive amount is not beneficial from an economic perspective, because it results in a zero rather than positive payoff. It follows that rejections are motivated by psychological and social motivations. In particular, we hypothesize that feelings of being treated unfairly and being disappointed by the offer may lead to rejections. Such feelings are likely to be stronger when the offer is lower, and when the responder's expectations about the amount he will receive are higher. This leads to our next double hypothesis:

*H6a: The probability of rejection will be a decreasing function of the offer amount*.

*H6b: The probability of rejection will be an increasing function of the responder's expectation about the offer amount*.

When a responder is asked about the minimal amount that he believes that others will accept, he may think that others behave the same way he will, or he may perceive some differences between himself and others. But even if the intentions attributed to others are unequal to the responder's own intentions (regarding the minimal acceptable amount), we believe that the two are positively correlated. It follows that when a responder attributes a higher minimal acceptable amount to others he also has a higher acceptance threshold himself, leading to our last hypothesis:

*H7: The probability of rejection will be an increasing function of the responder's belief about the minimum that other responders will accept*.

## Results and discussion

### Distribution of beliefs and offers

With a total of 34 proposers and 34 responders in five periods, the data includes 170 offers and 170 accept/reject decisions. Accordingly, we obtained 170 samples for each belief variable that was elicited. The distribution of some main variables of interest over all periods is presented in Table 1.

**Table 1:** **Distribution of beliefs and offers**.

	**0–10%**	**11–20%**	**21–30%**	**31–40%**	**41–50%**	**51–60%**	**61–70%**	**71–80%**	**81–90%**	**% Round**
Amount proposers offer to responders	9	14	17	45	75	8	1	1	0	82
Amount responders believe they will be offered	1	1	13	66	84	4	0	1	0	94
Minimum amount proposers believe responders will accept	13	11	22	68	44	5	6	1	0	85
Minimum amount responders believe other responders will agree to accept	25	33	31	57	22	0	1	1	0	87
Responders' earnings	31	8	10	36	75	8	1	1	0	86
Proposers' earnings	30	1	1	5	60	52	12	8	1	89

*The table presents the number of observations in each range. The total of each row (without the “% round” column) is 170. The right column designates what percentage of the entire range of values for the variable in this row have a round percentage of the pie (ending with 0, i.e., 10, 20, 30, …, 90%); this is equivalent to Shekel amounts ending with 5 or 0*.

We can see in Table 1 that the amount offered is usually (120 out of 170 offers) between 31 and 50% of the pie (57 are of equal split, i.e., exactly 50%). Offers of more than 50% of the total pie (i.e., more than 25 Shekels) are uncommon (10 out of 170 offers). Of the 170 offers, 103 offers give the receiver less than 50%; 23 give only 20% or less. The distribution of responders' expectations about how much they will be offered is somewhat similar, but more condensed, and with a little higher mean. In 150 out of 170 cases they expect offers of 31–50%. They predict high offers (51% and above) less frequently than they occur (5 vs. 12 cases), but also under-predict low offers of up to 20% (2 such predictions compared to 23 such offers).

The minimum proposers expect responders to accept is infrequently higher than 50%, with the most prevalent range being 31–40%, and then the equal split or a little less (41–50%). A substantial number of observations are in the lower ranges up to 30%, and even in the range 0–10% we have 13 observations. Interestingly, when asked about what other responders will do, the responders become even more extreme than the proposers in anticipating low values of acceptable offers. 58 responders think that other responders will agree to offers of 0–20% (compared to 24 proposers in that range). Earnings are most often equal or with a low inequality in favor of the proposer. Some responders get low offers and nevertheless accept, resulting in 18 observations with responders' earnings between 11 and 30%. The 30 observations with proposers' earnings between 0 and 10% are all the result of rejected offers. There are 31 responders with earnings between 0 and 10% because in addition to the 30 rejections there was one responder who received an offer of 10% and accepted it.

Some more information is provided by looking at some relevant correlations. The correlation between the minimum proposers believe that responders will accept and the offers they give them is 0.386. The correlation between the minimum responders believe that other responders will accept and the amount responders think they will be offered is 0.384.

### Choices of round numbers

As reported in the right column of Table 1, the data have a large percentage of round choices, defined as amounts that end with 5 or 0 (since the pie to be divided is 50 Shekels, these amounts are 0, 10, 20%, … of the total pie). If choices followed a uniform distribution, then the percentage of round choices should have been around 22%^9^. Because the equal split (25) is a round number and is a frequent choice, it raises the percentage of round numbers, but it is only partially responsible for the right column including numbers of 82% and above, rather than numbers slightly above 20%. We think that this strong preference for round numbers has some interesting potential reasons and implications. One possible reason is that choosing non-round numbers may be perceived as small-minded, and people want to avoid feeling small-minded, or avoid being perceived as small-minded by others who observe their behavior. Another possible reason is that people find it easier to think about round numbers, and therefore choosing round numbers reduces their cognitive burden in these decisions and therefore is preferred. This behavior has implications for experimental design. For example, if adding a full range of choices for the subject rather than just a few round choices is very complicated, it may be reasonable to go with the simpler design because most subjects are likely to choose the round-number options anyway.

### Experimental decisions, beliefs and outcomes by period

Table 2 presents the means of various variables in the different periods. In line with previous research, the amount proposers offer to responders (*M* = 40.47%, *SD* = 13.13) is significantly lower than the amount proposers leave for themselves (*M* = 59.53%, *SD* = 13.13), a statistically significant difference using a *t*-test (*t* = 9.45, *p* < 0.001). Overall, 140 of the 170 offers were accepted. All offers above 40% are accepted, most offers of 40% are accepted (34 out of 38), and often lower offers are also accepted (e.g., 10 offers of 30% and 8 offers of 20%). It is therefore not surprising that on average, the responders' earnings (*M* = 36.27%, *SD* = 19.14) are lower than the proposers' earnings (*M* = 46.08%, *SD* = 23.26), a significant difference (*t* = 9.447, *p* < 0.001).

**Table 2:** **Experimental decisions, beliefs and outcomes by period**.

	**Mean % Period 1**	**Mean % Period 2**	**Mean % Period 3**	**Mean % Period 4**	**Mean % Period 5**	**Overall Mean %**	**Overall SD**
% Of the pie proposers offer to responders = A	45.70	40.00	40.06	40.00	36.58	40.47	13.13
Minimum % of the pie proposers believe responders will accept = B	38.88	40.30	38.24	38.94	37.36	38.74	14.37
Proposers' offer above belief = A – B	6.82	−0.30	1.82	1.06	−0.78	1.73	
% Of the pie responders believe they will be offered = C	43.42	45.30	43.76	44.12	44.94	44.31	7.78
Responders' belief above offer = C – A	−2.28	5.30	3.70	4.12	8.36	3.84	
Minimum % of the pie responders believe other responders will agree to accept	30.18	31.18	31.42	30.12	30.52	30.68	14.65
Responders' earnings (% of the pie)	42.24	36.00	36.58	35.94	30.58	36.27	19.15
Proposers' earnings (% of the pie)	43.06	46.36	48.70	49.36	42.94	46.08	23.26
Number of rejected offers	5	6	5	5	9		

Looking at the changes between periods, we can see that the three belief variables (minimum % of the pie proposers believe responders will accept; % of the pie responders believe they will be offered; and minimum % of the pie responders believe other responders will accept) do not fluctuate much and do not show a consistent time trend. The amount proposers offer to responders decreases from 45.70% in period 1–40% in period 2 (*t* = 2.32, *p* = 0.022), stays essentially constant until period 4 (between periods 2–3: *t* = −0.02, *p* = 0.980; between periods 3–4: *t* = 0.03, *p* = 0.976), and then drops again from 40% in period 4–36.58% in period 5 (*t* = 1.80, *p* = 0.075). The mirror picture of this is seen in the amount proposers leave for themselves, since the two amounts always sum to 100%. We can also see that the difference between offers and the proposers' beliefs about the minimal acceptable offer goes down from 6.82% in period 1 to −0.30% in period 2 (*t* = 1.62, *p* = 0.110) and later is relatively stable (the *p*-values of the *t*-test for difference in means between periods 2–3, 3–4, and 4–5 are also above 0.57).

The decrease in offers after period 1 could reflect the result that most offers were accepted (29 of 34), leading proposers to update their beliefs about the minimal acceptable amount downwards and consequently to offer less in the next period. However, here the experimental design, which elicited not only offers but also beliefs, becomes useful. Looking at the proposers' beliefs about the minimal acceptable amount, we do not see any downward change (the average increases from 38.88 to 40.3% between period 1 and 2). Moreover, if a small number of rejections should result in the proposers trying to offer less, it is not clear why there is no such trend after periods 2 and 3, in which the number of rejections is similar to that in period 1.

When we take periods 2–4 and compare them to period 1, the lower offers and stable rejections result in increased earnings for the proposers and reduced earnings for the responders. Between periods 4 and 5 proposers again lower their offers (from 40 to 36.58%). In period 5, as opposed to the reduction in offers after period 1, this results in an increase in the number of rejections to 9. The responders are then hurt by both the low offers and the increased rejections and earn much less than in any other period (about 15% less than in periods 2–4 and about 28% less than in period 1). The proposers earn in period 5 less than in periods 2–4, but similar to period 1 (because the reduced offers are offset by the increased rejections).

### Proposers' offers vs. expectations

We continue to test the hypotheses presented in the previous section, starting with proposers' consistency between expectations and actions and using individual-level data. One possible reason for offers that exceed the proposer's belief about the minimal acceptable offer (we have 75 such offers out of 170) is fairness considerations of the proposer. For example, the proposer may think that an offer of 30% will be accepted, but nevertheless offer 50% because she wants to be fair. This is in line with results in the dictator game, where in most cases the dictators give positive amounts to the other player despite him not being able to reject the split. A second possible reason for offers above the expected minimal acceptable amount is heterogeneity of responders. For example, the proposer may believe that the average responder requires 30%, but she may also think that some responders will accept only offers above 40%, and decide that there are sufficiently many of these to justify offering 40% to increase the likelihood that the offer will be accepted. Such behavior of increasing offers to reduce the risk of rejection, at a cost of reducing the proposer's payoff if the offer is accepted, is likely to be more prominent the more risk averse is the proposer. This is because a higher offer means a lower possible payoff (in case of acceptance) but with more certainty.

In 59 offers, the amount offered was exactly equal to the proposer's belief about the minimum the responder would accept, a reasonable behavior if the proposer wants to give the minimum that is required to get her offer accepted. However, it is harder to justify the 36 offers in the data that are below the proposer's belief about the minimal acceptable offer. If a proposer believes that an offer below 30% will be rejected, then offering lower amounts is likely to be rejected and yield her a zero payoff. Why would she then offer such a low amount? We therefore refer to such decisions as “inconsistent,” whereas “consistent” decisions are those where offers are not lower than the proposers' beliefs about the minimal acceptable offer. The number of inconsistent decisions is equal to 7, 8, 5, 8, and 8 in periods 1–5, respectively; it varies only a little between periods and does not show any clear time trend.

One possible reason for offers below the expected minimal acceptable amount is that some proposers may feel that the responders' minimal acceptable amount is too high and unjustified. These proposers may then prefer to take the risk of rejection than to offer more than they think the responder deserves. Another possible reason for inconsistent decisions is that the proposer believes that there is enough variation in the responders' threshold for acceptance that it is worth trying to keep a larger share of the pie despite the increased chance of rejection^10^. This suggests once again that the behavior of proposers may be related to their risk attitudes. However, the inconsistent decisions may also represent mistakes or inconsistency of the proposer between her response to the belief question and the decision about the offer to make.

Next, we test consistent and inconsistent decisions separately. The average “proposers' offer above belief” computed only for the 134 cases in which it is zero or positive (i.e., consistent decisions) is 6.94%. It starts at 12.38 in period 1 and drops to the range 5.24–6.00% in the next four periods. The initial decline is mostly due to the decrease in amounts offered after period 1. For the 134 consistent decisions, a regression where the offer is the dependent variable and the belief about the minimal acceptable offer is the independent variable gives a coefficient of 0.69 (*p* = 0.000). For the 36 inconsistent decisions (negative “proposers' offer above belief”) there is no time trend, and the average value is −14.58, −20.76, −18.40, −14.24, and −20.26% in periods 1–5, respectively.

**Figure 1:**
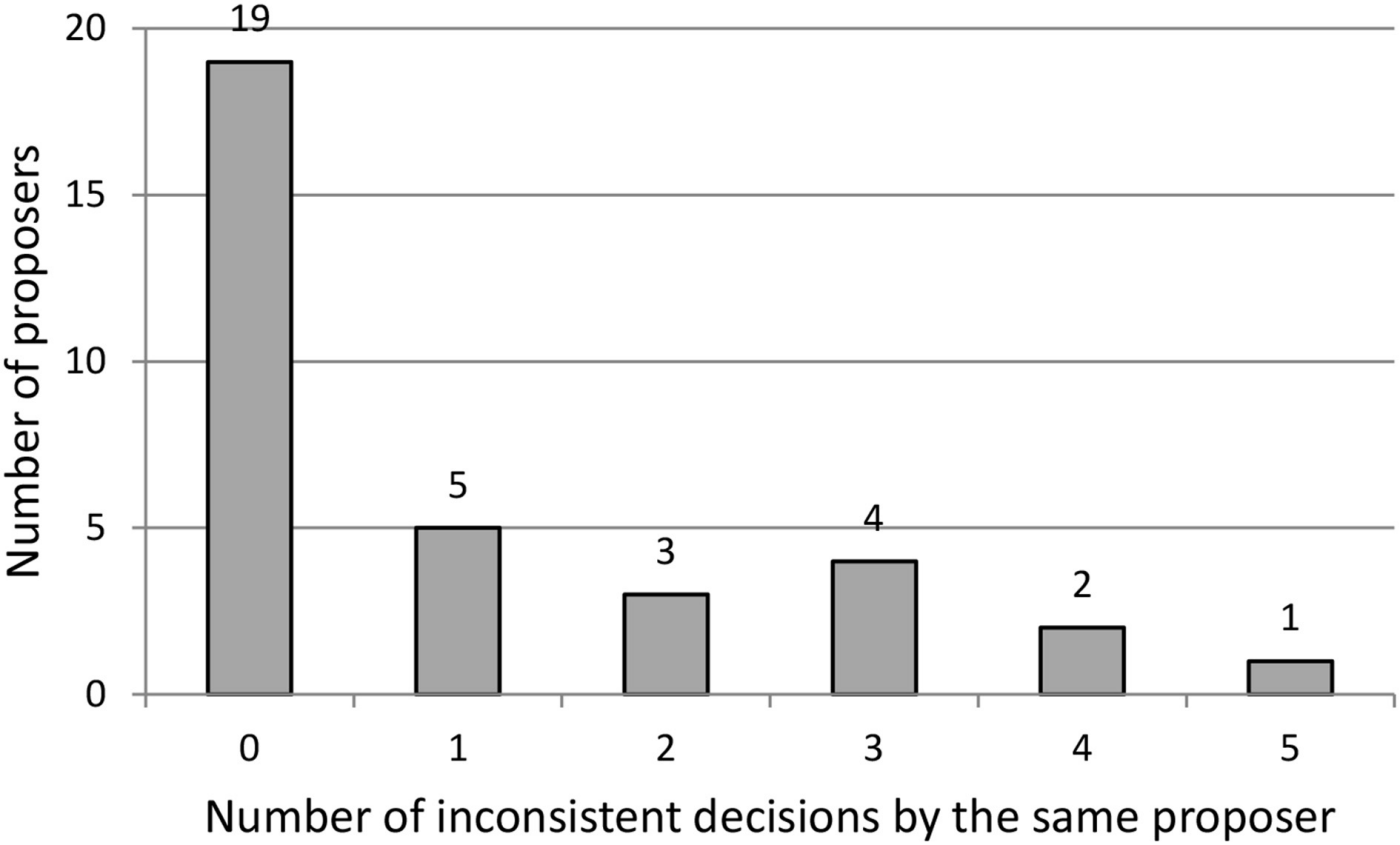
**Number of inconsistent decisions by the same proposer**.

Are people “consistently” inconsistent? To answer this question we count the number of inconsistent offers for each subject (recall that each proposer makes five offers during the experiment). Figure 1 presents the results. Of the 34 proposers, 15 are inconsistent at least once, and 10 make more than one inconsistent decision. Only one proposer is inconsistent in all rounds. Thus, a substantial number of proposers (although not a majority of them) violate our hypothesis *H1*.

### Accuracy of expectations

We now move on to test hypothesis *H2*. As presented in Table 2, the amount responders expect to receive is 44.30% on average, whereas the average offer is 40.48%. The difference is statistically significant (*t* = 3.30, *p* = 0.001). Considering the difference between expectations and offers in each period separately (see “Responders' belief above offer” in Table 2) shows an interesting switch from a negative difference in period 1 to positive in the other periods. The amount responders expect to receive fluctuates relatively little, so the main changes in this difference, after periods 1 and 4, are due to the decrease in the offers. When considering the periods separately, in some periods the difference is not statistically significant (recall that each period has only 34 observations of the offer and the responder's expectations). In periods 1, 2, 3, 4, and 5 the *p*-value of the *t*-test for difference in means between the offer and the responders' expectations is 0.389, 0.094, 0.182, 0.038, and 0.001, respectively. Thus, with the exception of period 3, each period has a lower *p*-value than the preceding period. This is surprising. A reasonable conjecture would yield the opposite pattern: as responders accumulate experience in the game they should become better in predicting the offer, and the difference between offers and expectations should then become less significant over time. The data, however, show that responders' expectations at the aggregate level do not seem to be updated much based on the new information in each period. In periods 2, 3, and 4 responders get an average offer of about 40%, and yet their average expectation hardly changes, remains on average a little above 44%, and even goes a bit up after periods 3 and 4. This pattern may be interpreted as overconfidence of responders in their beliefs, a phenomenon that was illustrated in other contexts (e.g., Barber and Odean, 2001; Malmendier and Tate, 2005). Overall, we see that the data do not support *H2* and the responders' expectations regarding the offers are not accurate on one hand, and not pessimistic and lower than actual offers on the other hand, but rather they tend to be optimistic.

Moving to hypothesis *H3*, we see in Table 2 that the minimum amount proposers believe that responders will accept (*M* = 38.74%, *SD* = 14.36) is higher than the minimum amount responders believe will be accepted by other responders (*M* = 30.68%, *SD* = 14.66), a significant difference (*t* = 5.12, *p* < 0.001). This implies that our hypothesis *H3* is not supported by the data. What creates this significant difference in expectations between proposers and responders? This is an interesting question but to answer it further research will be required.

### The effect of rejections on beliefs and offers

To test Hypothesis 4, we define “Rejected t-1,” a dummy variable that equals 1 if the offer in the previous period was rejected and 0 otherwise. This variable is only defined for proposers in periods 2–5. We also define “Proposer belief” as the proposer's belief about the minimal acceptable offer; “Offer” is the amount offered by the proposer to the responder; “Period” is the period number (an integer between 1 and 5); and “Female” equals 1 for females and 0 otherwise. Period and Female are added as control variables and can detect if a significant time trend or gender effect exists. We denote the lagged variables (i.e., these variables in the previous period) by adding “t-1.”

To analyze how rejection affects beliefs, we estimate Regression (1), presented in Table 3. Hypothesis 4a suggests that following a rejected offer, the proposer's belief about the minimal acceptable offer will increase. Regression (1) shows that this is not the case in the data. The coefficient of “Rejected t-1” that was supposed to be positive according to *H4a* is in fact negative and not statistically significant. As a robustness check, we also look at an alternative dependent variable, which is the change from period t-1 to period t in the Proposer's belief. *H4a* would predict that the coefficient of Rejected t-1 should have a positive effect on this new dependent variable. Regression (2), however, reveals that the coefficient is negative, very close to zero, and not statistically significant. Thus, the two regressions do not support *H4a*. Receiving a rejection does not increase the subject's belief about the minimal offer that will be accepted. This is surprising, especially if we remember that in most cases offers are above the proposer's belief about the minimum that the responder will accept (henceforth we sometimes use “Responder WTA” as a shorthand for the minimal amount responders will be willing to accept, with WTA standing for “willingness to accept”). It is intriguing why a rejected offer does not make the proposer revise her beliefs. It seems that the proposers are so confident in their beliefs that they do not easily change them. However, given that subjects are not experienced in this game, it is reasonable to update beliefs based on the observed behavior of responders. The finding that proposers do not do so may be another example for overconfidence, similar to our findings about the responders' inflexible expectations.

**Table 3:** **Regression results**.

	**Regression (1)**	**Regression (2)**	**Regression (3)**	**Regression (4)**	**Regression (5)**
**Dependent variable**	**Proposer belief**	**Proposer belief change from t-1 to t**	**Offer**	**Offer change from t-1 to t**	**Change in responder's expectation from t-1 to t**
**INDEPENDENT VARIABLE**					
Constant	9.24 (0.127)	1.64 (0.132)	17.24 (0.006)	−6.87 (0.028)	0.05 (0.948)
Rejected t-1	−2.03 (0.515)	−0.18 (0.933)	5.99 (0.103)	14.83 (0.001)	
Proposer belief t-1	0.82 (0.000)				
Offer t-1			0.53 (0.000)		
Offer above expectation in *t-1*					0.16 (0.018)
Period	−0.66 (0.030)	−0.62 (0.038)	−0.11 (0.858)	0.72 (0.274)	0.16 (0.639)
Female	−0.37 (0.755)	0.51 (0.537)	−1.37 (0.596)	−0.58 (0.734)	0.47 (0.480)
R^2^	0.71	0.01	0.28	0.18	0.16
N	136	136	136	136	136
Wald χ^2^	278.90	4.97	112.10	10.63	8.16
Prob > χ^2^	0.0000	0.1741	0.0000	0.0139	0.0429

*p-values are reported in parentheses. All regressions are GLS. The models are clustered by session. “Rejected t-1” equals 1 if the offer in the previous period was rejected and 0 otherwise. “Proposer belief” is the proposer's belief about the minimal acceptable offer and “Offer” is the amount offered by the proposer to the responder. Both beliefs and offers are in percentage of the pie, i.e., between 0 and 100. “Period” is the period number and “Female” equals 1 for females and 0 otherwise*.

Following the results above it is natural to expect that *H4b* will also not be supported by the data. The hypothesized mechanism was that a rejection causes the belief about the Responder WTA to increase, and this also increases the next offer. But we now see that the first step in this chain is missing, because proposers' beliefs do not change after a rejection. However, Regression (3), in which the dependent variable is the actual offer, shows that a rejection increases the next offer by about 6% of the pie compared to an acceptance. Of the 170 offers, 113 are between 40 and 50%, and among the 140 accepted offers, 109 are between 40 and 50%. So an increase of 6% of the pie is economically meaningful given that much of the variation in the data is in a 10% range. As a robustness check, we also consider in Regression (4) an alternative dependent variable, the change from period t-1 to period t in the Proposer's offer. The results are even more salient than in Regression (3), with a coefficient of 14.83 and *p*-value of 0.001. This suggests that our hypothesis *H4b* is supported by the data.

**Figure 2:**
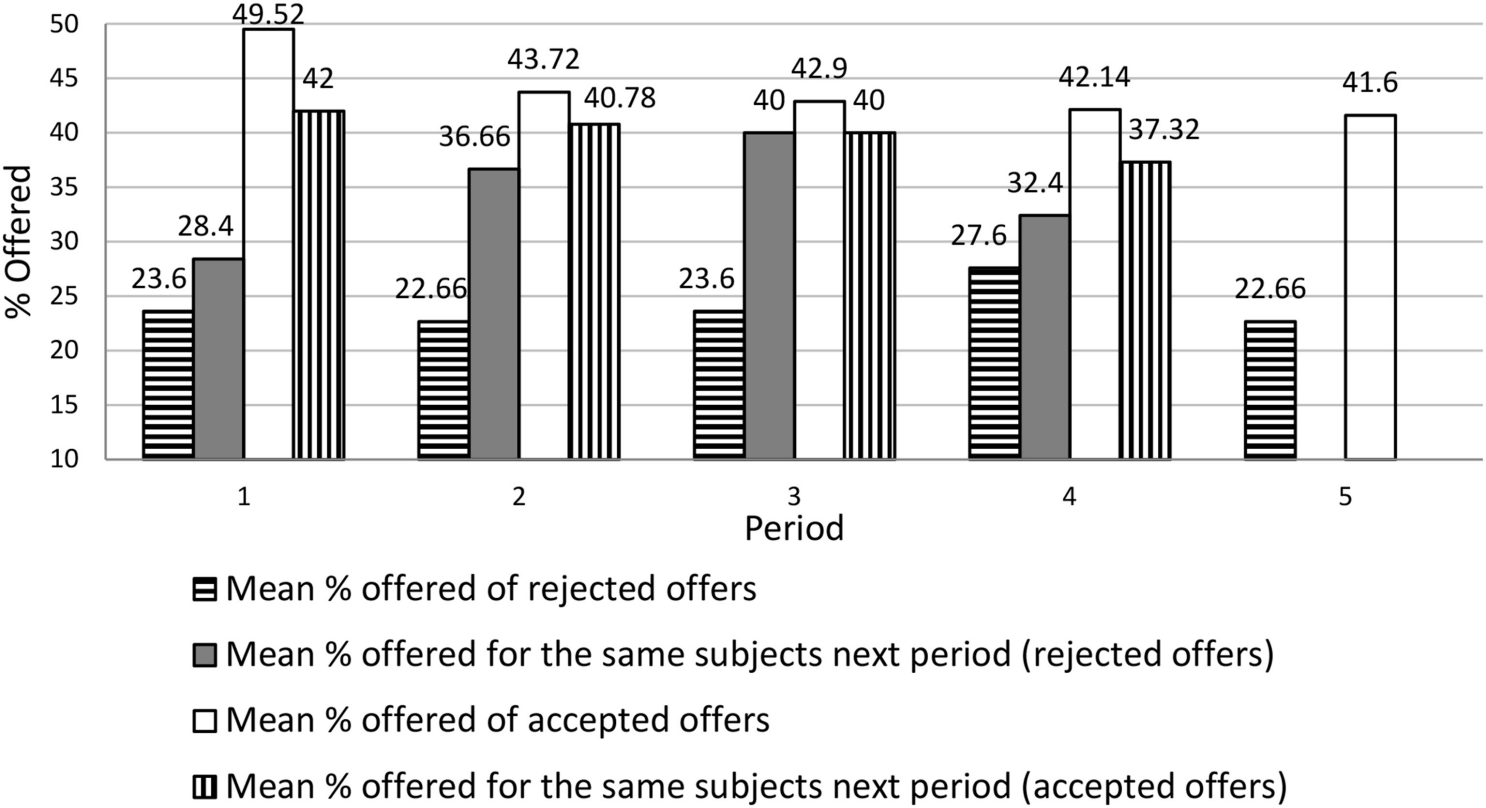
**Rejected versus accepted offers in the next period**.

Figure 2 presents a more detailed look at the effect of rejection by comparing in each period the average offer for the rejected vs. accepted offer. Then, we take the same subjects and analyze their offers in the next period. We see clearly the effect of rejection on the next offer. For accepted offers, the average always goes down (by 2.90–7.52% in different periods). For rejected offers, the average always goes up (by 4.8–16.4%).

The results are puzzling. We hypothesized that a rejection will lead the proposer to raise her belief about the Responder WTA, and then consequently raise her next offer. We see the next offer being raised but not the belief itself. If the belief is not changed, why does the offer increase? A possible explanation is that the proposers remain confident about the average level of the Responder WTA, but raise their expectations about its variance; that is, the rejection causes them to think that heterogeneity among responders is higher than they previously thought. Then, even if the average Responder WTA is believed to stay the same, a higher offer is justified because it more often (than when Responder WTA heterogeneity was assumed to be lower) avoids the situation in which the responder has a Responder WTA above average and will reject the offer^11^.

### The dynamics of responders' expectations between periods

To address hypothesis *H5*, we compute “expectations difference,” defined as the amount a responder expects to receive in the current period minus the amount he expected in the previous period. We also compute the responder's offer above belief in the previous period, defined as the amount offered to him in the previous period minus the amount he expected to receive in the previous period (all these offers and expectations are measured in percentage of the pie). A regression with the expectations difference as the dependent variable and the offer above responder's expectation in the previous period as an independent variable [see Regression (5) in Table 3] gives a coefficient of 0.16 for the latter (*p* = 0.018). This positive coefficient is in line with our hypothesis *H5* and it suggests that the responder's beliefs about what he is likely to be offered is not fixed but rather is shaped by previous outcomes in the game. The coefficient being only 0.16, however, also shows that the adaptation of responders' expectations is very partial: the responder updates his belief by only about one sixth of his mistake in the previous period. The low adaptation is probably the reason that at the aggregate level we do not observe convergence of expectations to actual offers (see the discussion of hypothesis *H2*).

### The probability of rejection

To analyze the last two hypotheses we estimate regressions where the dependent variable—“Rejected”—is equal to 1 if the responder rejected the offer and 0 if he accepted. For the sake of robustness, we estimate both the GLS linear probability model (LPM) and a logit model. “Responder Expectation” is the amount the responder expected to receive, whereas “Belief about Others” is the minimal amount the responder thought that other responders will accept. Regressions (6) and (7) in Table 4 show that hypotheses *H6a* and *H6b* are strongly supported by the data; the relevant coefficients have *p*-values below 0.01 in both regressions. As we hypothesized in *H6a* and *H6b*, the probability of rejection is a decreasing function of the offer and an increasing function of the responder's expectation about the offer. Regression (6) suggests that increasing the offer by 1% of the pie decreases the probability of rejection by about 1.7%. An increase of 1% of the pie in the responder's expectation about the offer increases the probability of rejection by about 0.7%. Thus, the offer amount is more important than the responder's expectations in its impact on the probability of rejection. The impact of the offer is more obvious and can be observed also in other ultimatum game studies. However, eliciting expectations is more unique to our study and the significant impact of expectations is interesting. Our interpretation of it is that the expectations reveal the responder's attitude about what a fair and appropriate offer is. Moreover, a given offer is more disappointing the more it is below the expectations. Therefore, the psychological reaction to a given offer also depends on expectations and consequently expectations also affect the probability of rejection. We also see that women are less likely to reject offers than men, by about 10%, a result that is statistically significant at the 5% level. The results of Regression (7), which uses the Logit model, are qualitatively similar, except for the gender effect that goes in the same direction but here it is no longer statistically significant.

**Table 4:** **Analyzing the responder's decision whether to reject the offer**.

	**Regression (6) GLS—LPM**	**Regression (7) Logit**	**Regression (8) GLS—LPM**	**Regression (9) Logit**
**Dependent variable**	**Rejected**	**Rejected**	**Rejected**	**Rejected**
Constant	0.649 (0.000)	−0.806 (0.570)	0.790 (0.000)	1.378 (0.206)
Offer	−0.017 (0.000)	−0.148 (0.000)	−0.017 (0.000)	−0.162 (0.000)
Responder expectation	0.007 (0.007)	0.108 (0.002)		
Belief about Others			0.005 (0.001)	0.091 (0.005)
Period	−0.013 (0.374)	0.015 (0.929)	−0.010 (0.498)	0.055 (0.800)
Female	−0.102 (0.036)	−1.347 (0.110)	−0.081 (0.005)	−1.401 (0.009)
R^2^ (Pseudo R^2^for Logit)	0.38	0.43	0.40	0.48
N	170	170	170	170
Wald χ ^2^	114.52	75.6	157.29	126.36
Prob > χ ^2^	0.0000	0.0000	0.0000	0.0000

*p-values are reported in parentheses. The models are clustered by session. “Rejected” equals 1 if the offer was rejected and 0 otherwise. “Offer” is the amount offered by the proposer to the responder. “Responder Expectation” is the amount the responder expected to receive, and “Belief about Others” is the minimal amount the responder thought that other responders will accept. Both beliefs and offers are in percentage of the pie, i.e., between 0 and 100. “Period” is the period number and “Female” equals 1 for females and 0 otherwise*.

Regressions (8) and (9) address hypothesis *H7*. We still use the offer, period and gender as control variables, but now, “Responder Expectation” is replaced with “Belief about Others.” Both regressions support *H7*: attributing to others a higher threshold for acceptance raises the probability of rejection. The LPM model in Regression (8) suggests that an increase of 1% of the pie in “Belief about Others” raises the rejection probability by about 0.5%. Our interpretation is that the responder's belief about the acceptance threshold of other responders is positively correlated with his own threshold, and therefore creates a positive coefficient for “Belief about Others.” The results in the Logit model reported in Regression (9) are qualitatively similar to those of Regression (8). Both regressions also replicate the results of regressions (6) and (7) about a negative coefficient for “Offer” (which is statistically significant) and a negative coefficient for “Female” [which is statistically significant at the 1% level in regressions (8–9)].

Table 5 presents another look at the rejected offers, showing the number of rejected offers given the offered amount and the percentage responders believed they would be offered. We can see that all rejections come from cases in which the offer was smaller than the responder's belief about how much he will be offered. Most rejections occur when the responder expects to receive 50% of the pie and gets less. Offers above 40% were never rejected in our sample.

**Table 5:** **Frequency of rejection by offer and belief**.

	**Percentage responders believe they will be offered**						
	**20**	**40**	**46**	**50**	**54**	**60**	**Total**
Offer (%)							
0	1	1	0	0	0	0	2
2	0	1	0	1	0	0	2
10	0	2	0	2	0	0	4
20	0	2	0	3	0	1	6
26	0	1	0	0	0	0	1
30	0	1	1	4	0	0	6
34	0	0	0	1	0	0	1
36	0	0	0	1	0	0	1
38	0	1	0	2	0	0	3
40	0	0	0	3	1	0	4
Total	1	9	1	17	1	1	30

*The table shows the number of rejected offers given the offered amount and the percentage responders believed they would be offered. Only values for which some rejected offers exist are presented*.

## Conclusions

We take one of the important tools of behavioral economics, the ultimatum game, and add to the usual decisions about what offers to make and whether to accept them also an elicitation of beliefs and expectations. This, together with a multi-period game that allows to analyze changes due to experience, yield some interesting findings. We observe a strong preference for round choices (10, 20%, etc.) in offers and beliefs. Surprisingly, we find a substantial number of offers (36 of 170) that are below the proposer's belief about the minimal acceptable offer. We also find that responders do not predict accurately, even on average, the amount that will be offered to them, and do not get better in their predictions as they accumulate experience. At the individual level, we see some effect of the mistake in expectations in the previous period on the responder's expectation about the offer in the current period, but this effect is relatively small and consequently at the aggregate level we observe “sticky predictions” that hardly change.

We find that the proposers' beliefs about the minimum amount that responders will accept is significantly higher than the minimum amount responders believe will be accepted by other responders. This intriguing finding calls for additional research to understand its reasons. When we analyze how a rejection in one period affects the proposer's behavior in the next period, we observe an interesting and unexpected pattern. The proposer's belief about the minimal acceptable offer does not change, contrary to our predictions. Nevertheless, the proposer's offer in the next period does increase following a rejection. While we expect this result, the hypothesized reason for it was an increased belief about the minimal acceptable offer, so it is intriguing why we see the increase in offers without a change in beliefs.

When analyzing what affects the probability of rejection, we confirm our hypotheses. A higher offer decreases the probability of rejection whereas higher expectations of the responder about the amount that will be offered to him or higher beliefs about the minimal amount that other responders will accept increase the probability of rejection. The impact of the offer is the largest among these three variables. Females seem to reject offers less frequently, but this result is not always statistically significant.

The study illustrates how eliciting beliefs can add to our understanding of behavior in games and provides some interesting findings. In several cases we find some inconsistency between beliefs and actions. We also observe beliefs that are sticky and do not change much even in light of new evidence. We hope that this study will encourage others to continue this line of research and study how belief elicitation can contribute to our understanding of economic behavior in additional settings. In particular, we believe that some further studies of belief elicitation in ultimatum games may provide some interesting insights and also examine the robustness of the results reported here. One direction can be to conduct an experiment with more than five periods to examine time trends in decisions and beliefs over a longer horizon. Another direction can be to ask the responders to provide their decisions in the strategy method, i.e., to report what is the minimal amount that they are willing to accept. This can also enable to incentivize the belief elicitation procedure. Having different rules of matching (for example keeping the matching between the proposer and the responder fixed) or having role switching (proposers who later play as responders and vice versa) and analyzing how these variations affect behavior and beliefs may also provide some interesting insights. In addition, the proposer's behavior may also be influenced by risk attitudes (see García-Gallego et al. (2012) for the connection between risk attitudes and gender in the ultimatum game). Lower offers result in higher earnings for the proposer if accepted, but also increase the probability of a zero payoff due to rejection, and therefore more risk aversion can result in higher offers. Exploring the connection between beliefs, offers, and risk attitude of proposers in the ultimatum game may be an interesting direction for future research. Similarly, exploring the role of beliefs, their development over time, and their connection to decisions and risk attitudes can be interesting also in other strategic games where a player's payoff depends on his own decision as well as on other players' decisions, such as in the investment game (e.g., Buchan et al., 2008).

### Conflict of interest statement

The authors declare that the research was conducted in the absence of any commercial or financial relationships that could be construed as a potential conflict of interest.

## Footnotes

^1^If we assume that the responder accepts an offer when he is indifferent between doing so and rejecting it, the equilibrium involves a proposer who offers zero and a responder who accepts the offer.

^2^A notable exception in which many responders agreed to a zero offer (using the strategy method) is reported by Brañas-Garza et al. (2006), who conducted the ultimatum game in a gypsy community in Vallecas, Madrid.

^3^See chapter 2 of Camerer (2003) for a survey on dictator and ultimatum games and for an explanation regarding the contribution of the ultimatum game to studying social preferences. Another illustration of the importance of the ultimatum game and its large impact on understanding social behavior is provided by van Damme et al. (2014).

^4^The experiment conforms to the relevant regulatory standards, was approved by the Guilford Glazer Faculty of Business & Management at Ben-Gurion University of the Negev ethics committee, and informed consent was obtained from all subjects.

^5^Although in some sessions it was possible that participants were paired with the same person twice, they did not know if and when that happened and who that person was.

^6^The literature on this issue is wide and extensive. Two classic examples are Friedman (1983) and Selten (1998).

^7^Gächter and Renner (2010) report higher contribution levels in a public goods experiment when beliefs are incentivized. It is possible that participants considered the monetary incentive as some kind of compensation that allows them to contribute higher amounts. We believe that the circumstances in our experiment may be similar.

^8^However, recall that the proposer is re-matched with another receiver in each period. If a proposer believes that there is a distribution of receiver preferences, he may conclude after a rejected offer not that he was wrong in his belief about the mean of that distribution, but rather that he was just unlucky and sampled an extreme observation from this distribution (a receiver with a relatively high minimal acceptable offer). Then the proposer may infer that he need not update his beliefs about the minimal acceptable offer and need not change his offer in the next period. If the vast majority of proposers behave in this manner, then we should not find support for H4a and H4b in the data.

^9^There are eleven round numbers (0, 5, 10, …, 50) out of 51 possible ones (0, 1, 2, …, 50).

^10^A higher variation in responders' acceptance threshold around its average results in a smaller increase in the probability of rejection due to a given reduction in the offer (to below the average threshold).

^11^For example, if one thinks that Responder WTA is distributed uniformly in the range 16–22, and an offer of 22 gets rejected, a modified belief about a uniform distribution between 13 and 25 may justify an offer of 25 despite the fact that the assumed average of Responder WTA remains 19. In the question about the expected Responder WTA, the subject therefore may still answer the same way (19), but increase her offer from 22 to 25.
